# Left Atrial Appendage Closure with a New Occluder Device: Efficacy, Safety and Mid-Term Performance

**DOI:** 10.3390/jcm10071421

**Published:** 2021-04-01

**Authors:** Marc Llagostera-Martín, Hector Cubero-Gallego, Aleksandra Mas-Stachurska, Neus Salvatella, Andrea Sánchez-Carpintero, Helena Tizon-Marcos, Marcos Garcia-Guimaraes, Alicia Calvo-Fernandez, Luis Molina, Beatriz Vaquerizo

**Affiliations:** 1 Interventional Cardiology Unit, Cardiology Department, Hospital del Mar, 08003 Barcelona, Spain; marcllm91@gmail.com (M.L.-M.); hektorkubero@hotmail.com (H.C.-G.); amasstachurska@psmar.cat (A.M.-S.); nsalvatella@psmar.cat (N.S.); asanchezcarpintero@psmar.cat (A.S.-C.); htizon@psmar.cat (H.T.-M.); mgarciaguimaraes@psmar.cat (M.G.-G.); acalvofernandez@psmar.cat (A.C.-F.); lmolina@psmar.cat (L.M.); 2 Heart Diseases Biomedical Research Group (GREC), Hospital del Mar Medical Investigation Institute-IMIM, 08003 Barcelona, Spain; 3 Medicine Department, Autonomous University of Barcelona-UAB, 08193 Barcelona, Spain

**Keywords:** left atrial appendage closure, atrial fibrillation, stroke, LAmbre

## Abstract

The LAmbre^TM^ device is a novel system designed for left atrial appendage closure (LAAC). First registries showed a high rate of device implantation success. However, few mid-term results are available. We present our 1- and 12-month follow-up results for this device. This prospective, single-center registry included consecutive patients with nonvalvular atrial fibrillation who underwent LAAC with the LAmbre^TM^ device. Transesophageal echocardiography (TEE) was performed at 1-month follow-up. In total, 55 patients were included. The population was elderly (75 ± 9.4 years), with a high proportion of comorbidities. The mean CHA_2_DS_2_-VASc and HAS-BLED scores were 4.6 ± 1.6 and 3.9 ± 1.0, respectively. Previous history of a major bleeding event was present in 37 patients (67.3%). Procedural success was achieved in 54 patients (98.2%). Device success was achieved in 100% of patients in whom device implantation was attempted (54 patients). Major in-hospital device-related complications included mortality of one patient (1.8%) and pericardial tamponade in two patients (3.6%); the incidence of stroke was 0%. No thrombus or significant leaks (≥5 mm) were observed on 1-month TEE. At 12 months, adverse events were overall death (1.8%), transient ischemic attack/ischemic stroke (1.8%), and major bleeding events (Bleeding Academic Research Consortium (BARC) 3a and 3c; 11%). In this high-risk population, the LAmbre^TM^ device seems to be a safe and effective option for LAAC with a remarkable mid-term performance.

## 1. Introduction

Atrial fibrillation (AF) is the most prevalent arrhythmia in Europe [1]. Nonvalvular AF is associated with high risk of embolism and stroke, is the second most common cause of stroke, and increases morbidity and mortality in elderly patients [1,2]. Oral anticoagulation (OAC) is the standard treatment for stroke prevention, with a class I indication in patients with a ≥1 score (2 for women) on the CHA_2_DS_2_-VASc risk prediction scale [2,3]. In recent years, percutaneous left atrial appendage closure (LAAC) has become an alternative method for stroke prevention in patients with nonvalvular AF in whom OAC has failed or is contraindicated [4,5,6]. Several devices for LAAC have been commercialized since 2001. The first device, the PLAATO^TM^ (Appriva Medical Inc., Sunnyvale, CA, USA), was followed by other devices specifically designed to improve fixation, adaptability, and LAAC effectiveness [4,5,6,7,8]. Currently, the two most commonly used devices are the Watchman™ (Boston Scientific, Marlborough, MA, USA) and the Amplatzer Amulet™ (Abbott Vascular, Chicago, IL, USA). However, in some cases, the anatomy of the left atrial appendage (LAA) may hinder the implantation of these devices [9].

The LAmbre™ system (Lifetech Scientific, Shenzhen, China) is a new self-expanding and recapturable device specifically designed for LAAC including complex morphology. The first registries with the LAmbre™ showed a high device implantation success rate and remarkable short-term performance in terms of stroke prevention [10,11,12,13]. Nonetheless, the mid-term clinical data for this device are still limited.

The aim of this study was to provide additional information on the safety and effectiveness of the LAmbre™ device for LAAC in patients with nonvalvular AF by reporting the 1-month and 12-month follow-up results.

## 2. Materials and Methods

This prospective, observational, single-center study consecutively enrolled all patients who underwent LAAC with the LAmbre™ device (Lifetech Scientific, Shenzhen, China) between March 2017 and November 2019.

The inclusion criteria were age ≥18 years with a diagnosis of AF, contraindication for OAC, and high embolic risk (with a CHA_2_DS_2_-VASc score ≥ 2). The indication for LAAC was approved in all cases by a heart team based on current clinical practice guidelines and indications. The exclusion criteria were as follows: mechanical valve prosthesis, recent (<30 days) acute myocardial infarction or stroke, planned cardiac surgery, active endocarditis, and life expectancy < 1 year.

Patient, echocardiographic, and procedural characteristics, as well as adverse events, were collected from an electronic database with individual access.

This study was performed according to the tenets of the Declaration of Helsinki, ISO14155, and clinical practice guidelines. The study protocol was approved by the Institutional Ethics Committee and the hospital research committee. Informed consent was obtained from all patients.

**Figure 1:**
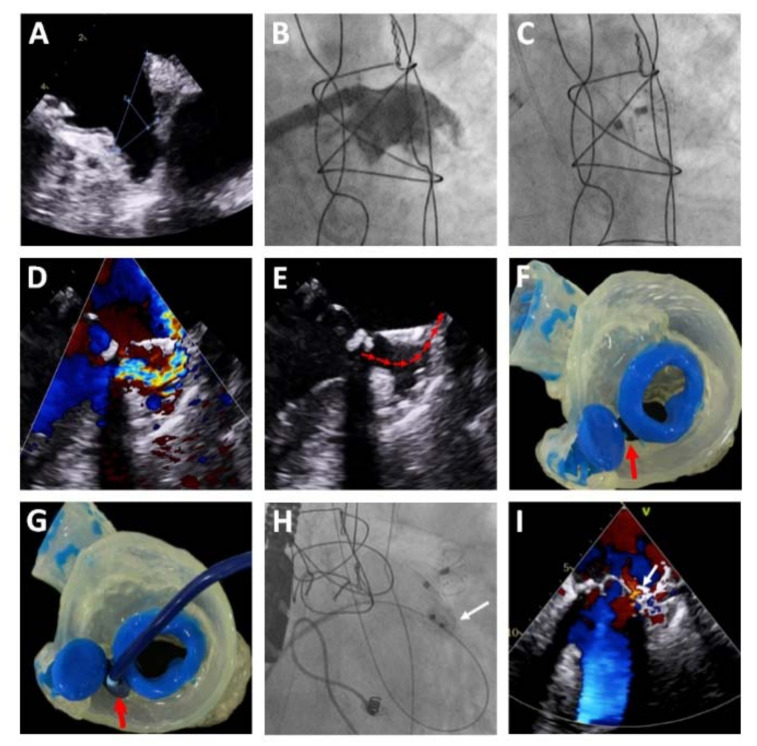
Case of left atrial appendage closure (LAAC) with the LAmbre™ device showing the resistance of the device to direct impact from a severe regurgitation jet from a mitral periprosthetic leak. Legend: (**A**–**C**) device implantation. (**D**,**E**) transesophageal echocardiography (TEE) showing a regurgitation jet from the leak toward the device, flowing between the cover disk and the umbrella (red arrow). (**F**,**G**) 3D model for planning of the percutaneous closure of the leak (red arrow). (**H**) Percutaneous closure of the leak with a 14 × 5 mm Amplatzer Vascular Plug III device (Abbott Vascular, USA) (white arrow). (**I**) TEE confirming the correct implantation of the device (white arrow) with no interference with the LAAC system or with the cusps of the mitral bioprosthesis, reducing the regurgitation to mild.

**Figure 2:**
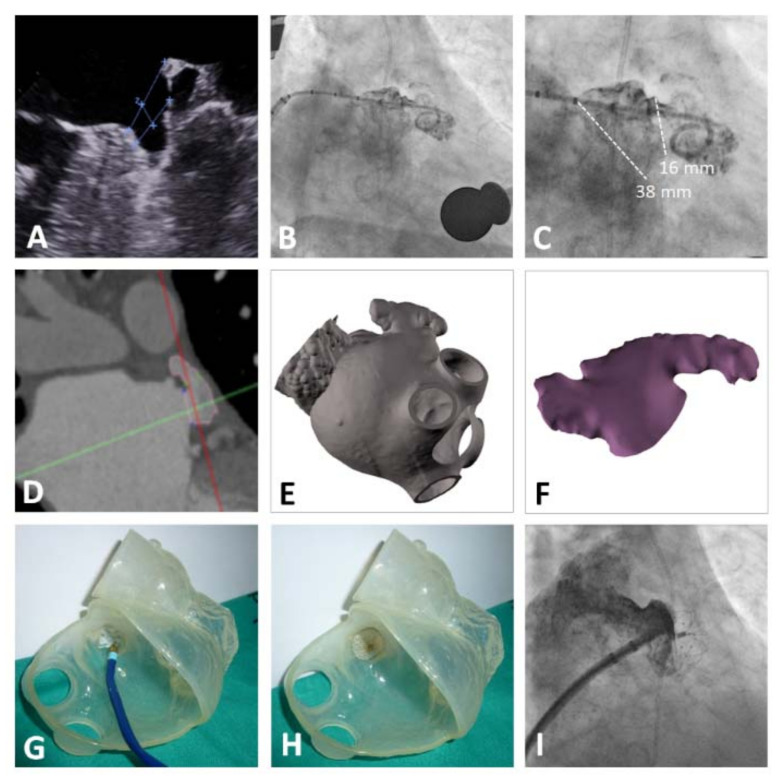
Small, slight, and multilobar LAAC. Legend: (**A**) TEE image of the LAA. (**B**,**C**) LAA angiography. (**D**) CT of the LAA. (**E**,**F**) Three-dimensional reconstruction of the LAA. (**G**,**H**) 3D-printed model to help with selection of the device size and plan the procedure. (**I**) Successful implantation of a 16-mm LAmbre™ device.

The LAmbre™ system comprises a self-expanding nitinol structure consisting of an umbrella and a cover disk connected by a short central waist [14]. The umbrella has eight claws, each ending in a hook that enables optimal anchoring inside the LAA, and an external polyethylene terephthalate membrane that allows adequate adaptation to the internal anatomy of the LAA. The cover disk is filled with polyethylene material that ensures complete sealing of the LAA orifice [14]. The design ensures that the device is sufficiently resistant to guarantee adequate anchoring to the LAA (Figure 1). The complex architecture of the device, together with the variety of sizes and the availability of models with oversizing of the cover disk versus the umbrella, permits optimal implantation of the device in atrial appendages with highly complex morphologies, such as multilobar atrial appendages (Figure 2).

Prior to the procedure, an anesthesia evaluation was performed, as well transesophageal echocardiography (TEE) to assess the presence of thrombosis, determine the morphology of the LAA, and obtain the measurements of interest: orifice diameter, landing zone, and maximum depth of the LAA at different angles (0°, 45°, 90°, 130°, and 180°). CT scan was performed in one case of atrial appendage with highly complex morphology (multilobar) to make the sizing, and in two cases due to the presence of esophageal varices. All procedures were performed by two experienced operators in the catheterization laboratory, under general anesthesia and guided by TEE, with assistance from an anesthesiologist and imaging expert. However, cases with contraindication for TEE were guided by intracardiac echocardiography.

A transseptal puncture was made in an inferoposterior location to ensure that the delivery catheter was parallel to the long axis of the LAA. LAA angiography was performed with a pigtail catheter in the right oblique (30°), cranial (10°), and caudal (10°) projections. The device size was chosen based on the measurements of the landing zone and LAA orifice obtained from the TEE and LAA angiography. The device was chosen to be 3- to 5-mm larger than the maximum diameter of the landing zone. After the angiography, a stiff guidewire was advanced into the LAA. The delivery catheter was advanced over this wire to deliver the occluder.

Once the device umbrella was deployed, its correct attachment was checked using a tug test under angiography and TEE. Before release, TEE was used to ensure that the cover disk did not interfere with the mitral valve apparatus and that adequate sealing of the orifice was achieved, based on the absence of peridevice leaks ≥5 mm. In the case of LAAs with complex, small, and/or shallow morphologies, to obtain adequate support, the guidewire was advanced to the left superior pulmonary vein and was exchanged for a pigtail catheter, which enabled safer insertion into the LAA. With the pigtail catheter within the LAA, the stiff guidewire was advanced and was followed by the delivery catheter over the guidewire–pigtail assembly, ensuring the stability of the system and reducing the risk of perforation (Figure 2). When the procedure was guided by intracardiac echocardiography, computed tomography was performed to help with the selection of the device size.

Patients were discharged 24 h after the procedure after transthoracic echocardiography ruled out device dislodgment, embolization, and pericardial effusion. At discharge, all patients were prescribed single antiplatelet therapy with aspirin for 3 months. TEE follow-up was performed 1 month after the procedure to assess the presence of leaks and/or thrombi. Clinical follow-up of the patients was performed at 1, 3, and 12 months. At the 3-month clinical follow-up, antiplatelet therapy was withdrawn if the patients had not experienced any embolic event and there was no other clinical reason for this treatment.

According to the Munich consensus document on the LAAC procedure [15], device success was defined as correct deployment and implantation of the device in the right position; procedural success was defined as adequate exclusion of the LAA, no peridevice leak ≥ 5 mm, and no complications [15].

Complete sealing of the LAA by the device, both in the immediate postprocedural period and at echocardiographic follow-up, was defined as the absence of peridevice leaks ≥ 5 mm at all angles on Doppler–TEE scanning.

Procedure-related events were those occurring in the first 72 h after implantation.

Vascular complications were considered to be the following: puncture site hematoma ≥6 cm, arteriovenous fistula, retroperitoneal hematoma, aneurysm, thrombosis, and arterial dissection, as well as the need for vascular surgery.

Bleeding events were described according to the Bleeding Academic Research Consortium (BARC) classification, with major bleeding considered those events classed as BARC 3a to 5 [16].

Statistical analysis was performed with IBM^®^ SPSS^®^ Statistics software version 25 (IBM, Armonk, NY, USA). Continuous variables are presented as mean ± standard deviation or as median (interquartile range). Categorical variables are expressed as frequencies and group percentages.

## 3. Results

This study included a total of 55 patients with nonvalvular AF who underwent LAAC with the LAmbre™ device between March 2017 and November 2019. Baseline clinical and procedural characteristics are detailed in Table 1 and Table 2. As shown in Table 1, the study included an elderly population (mean age, 75 ± 9.4 years) with a high proportion of patients with hypertension (94.5%), chronic kidney disease (74.5%; 12.7% on hemodialysis), diabetes mellitus (49%), chronic ischemic heart disease (32.7%), and previous stroke/transient ischemic attack (TIA) (38.2%) and with high embolic and bleeding risk (mean CHA_2_DS_2_-VASc and HAS-BLED scores, 4.6 ± 1.6 and 3.9 ± 1.0, respectively). Of the patients, 67.3% had previous history of major bleeding.

**Figure 3:**
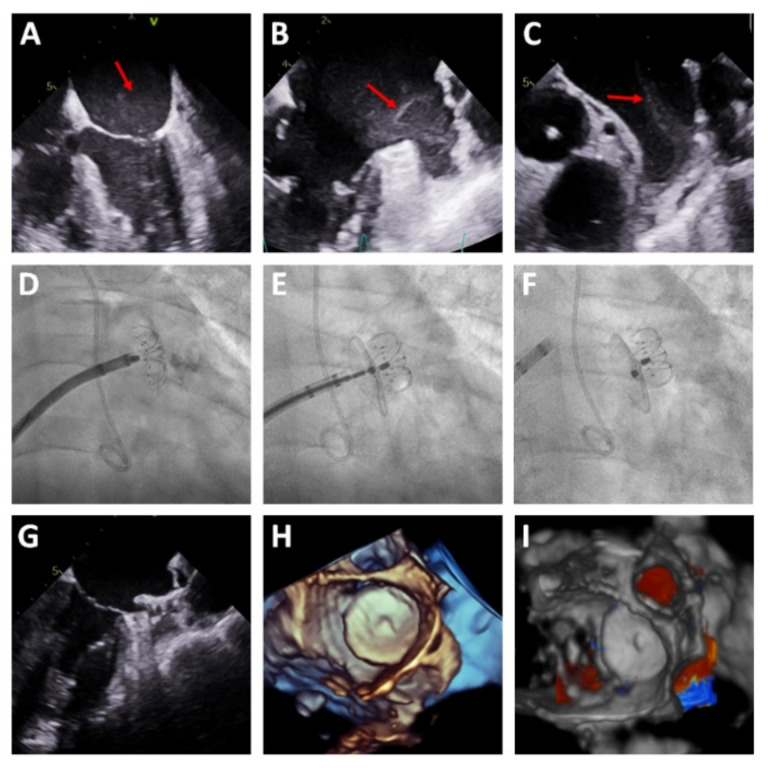
LAAC with early thrombosis. (**A**) TEE suggestive of “smoke” in the left atrium (red arrow). (**B**,**C**) Smoke/early thrombosis in the LAA (red arrow). (**D**–**F**) LAAC guided exclusively by TEE. (**G**) TEE verification of the correct implantation of the device. (**H**,**I**) 3D TEE showing the LAAC device without a peridevice leak.

Procedural characteristics are detailed in Table 2. Most procedures (94.5%) were performed under general anesthesia and TEE guidance. Per protocol, coronary angiography was performed before the LAAC in the same session in all patients with unknown coronary anatomy. In three patients, the LAAC was performed in the presence of early thrombosis in the LAA; in these patients, the procedure was guided by TEE alone, without contrast injection (Figure 3). Procedural success was achieved in 54 patients (98.2%) and no patients were excluded due to complex LAA morphology. Device success was achieved in 100% of patients in whom device implantation was attempted (54 patients). In one patient, the device implantation was not attempted due to cardiac perforation while performance intracardiac echocardiography, requiring emergency surgery.

The median length of hospital stay was 1 day. After the procedure, patients spent two hours in the recovery room before being transferred to the inpatient cardiology unit with telemetry.

Periprocedural characteristics are detailed in Table 3. One patient with chronic kidney disease on hemodialysis developed cardiac tamponade 6 h after the LAAC and was successfully treated with pericardiocentesis. After repeat TEE and CT, perforation of the LAA was ruled out; thus, the complication was attributed to inflammation related to the maneuvers required for device placement.

There was only one death: a high-complexity patient whose left atrium was perforated during the intracardiac echocardiography performed to guide the procedure (TEE imaging quality was insufficient to guarantee adequate LAAC with the device). Pericardiocentesis and emergency surgical suturing were successfully performed. Nonetheless, the patient died of multiorgan failure 48 h after the event.

No vascular complication requiring surgery occurred. There was only one early (<24 h) type 3a postprocedural bleeding event, related to the venous access (major bleeding), which did not require surgery. The initial rate of adverse events is shown in Table A1 of the Appendix A. After the first 27 cases, the rate of adverse events was 0% for the subsequent 28 cases.

TEE was performed at 1-month follow-up in 50 patients (92.6%). No CT scan was performed at 1-month follow-up. The follow-up TEE characteristics are shown in Table 4. There was no incidence of significant peridevice leak or thrombus formation on the surface of the device. TEE could not be performed in four patients due to contraindication related to the esophageal anatomy; follow-up was performed using transthoracic echocardiography, which showed correct apposition of the device and no jets suggestive of significant leak in any patient.

The 1-year clinical follow-up was completed in all patients. There was no loss to follow-up. The adverse events of these 54 patients at the 1-year follow-up are shown in Table 5. The overall mortality rate was 1.8% (1 patient). One patient (1.8%) had an ischemic stroke during follow-up. Six patients (11%) had a major bleeding event, one of which was a hemorrhagic stroke. The details of the patients who experienced major bleeding during follow-up are shown in Table A2 of the Appendix A. In all of these patients, the reason for the LAAC was major bleeding. The median time from closure to bleeding was 3.5 months (1–9 months). Of the six patients, five were under treatment with aspirin at the time of the major rebleeding. Only one patient was not under treatment with antiplatelet therapy.

## 4. Discussion

Several aspects of this prospective registry are worth highlighting. First, the immediate and 1-month follow-up results suggest that the LAmbre™ is one of the safest and most effective devices, with a device implantation success rate of 100% and with no exclusion of complex LAA morphologies. Second, this is the largest registry published with 1-year follow-up of this device showing remarkable mid-term performance regarding prevention of ischemic stroke at 1-year follow-up. Third, this is the first registry to include a single and short antiplatelet therapy with aspirin 100 mg/day for 3 months after LAAC with a 1-month rate of device thrombosis on TEE of 0%, lower than reported in other registries with more aggressive antiplatelet/OAC regimens.

Our patient cohort comprised patients with nonvalvular AF who were contraindicated for OAC and had high clinical complexity and the concomitant presence of high ischemic and bleeding risk. Compared with the registries of the Watchman™ and Amplatzer Amulet™ devices, the present study shows a higher percentage of patients with chronic kidney disease and with higher CHA_2_DS_2_-VASc and HAS-BLED scores [7,8,17,18,19]. In comparison with previous studies of the LAmbre™ system [10,11,12,14], this registry shows the same profile of high-complexity patients, although with higher CHA_2_DS_2_-VASc and HAS-BLED scores. The immediate results with this device are notable (100% device success) and in line with the few existing registries of LAAC with the LAmbre™ thus far published, with device success rates from 99.4% to 100% and procedural success rates from 92% to 99.4% [10,11,12,14].

A unique characteristic of this device is its wide variety of umbrella sizes, from 16 mm to 36 mm, with a cover disk 6-mm larger than the umbrella and with six special sizes (16–26 mm) related to the much larger cover disk (30–38 mm). This variety of sizes allows sealing of all LAAs regardless of complex anatomy. The need for an intraprocedural change in device size was low (5.4%) and lower than other registries [19].

Severe procedure-related complications included the death of one patient due to LAA perforation during intracardiac echocardiography performance, which required emergency surgery. Because we could not proceed with device implantation in this patient, it was not considered a device-related complication.

The incidence of other severe procedure-related complications (5.4%) was comparable to the previous studies performed with the same device [10,12]. The first study with the LAmbre™, carried out by Huang et al. [12], was an open-label, non-randomized study conducted in 12 centers in China, which included 152 patients and showed a major complication rate of 3.3%. The largest registry of LAAC with the LAmbre™ in Europe was carried out by Park et al. [10]. This open-label, non-randomized study, conducted in two German hospitals, included 60 patients and showed a major complication rate of 6.7%.

The two large studies with the Watchman^TM^ device, PROTECT-AF [20] and PREVAIL [21], showed complication rates of 8.7% and 4.5%, respectively. Notably, in our registry, there was no incidence of procedure-related stroke/TIA or device embolization. In addition, the last 28 patients of our registry had no complications, which appears to have been the result of both the learning curve of LAAC procedure and possibly the ease of device implantation.

The 1-month TEE follow-up (*n* = 50) showed no incidence of laminar thrombosis on the device surface. A systematic review that included a total of 30 studies of LAAC performed between 2008 and 2015 found an overall incidence of device thrombosis of 3.9% with a median time from implantation of 1.5 months (0.5–2.9 months) and rates of ischemic events of 2.4% for TIA and 4.9% for stroke [22].

In our study, no case of significant peridevice leak (≥5 mm) was detected or interference of the device with mitral valve function. The EWOLUTION study [17] reported a 1% rate of significant peridevice leaks with the Watchman^TM^ device. A study with the Amplatzer Amulet^TM^ device found a 1.9% rate of significant peridevice leaks [23].

Our follow-up results were noteworthy. Just one patient died during follow-up. An 85-year-old male patient died 8 months after the procedure due to frailty. One patient had an ischemic stroke during follow-up: a 73-year-old female with high comorbidity developed a clinical neurological deficit in the context of a hypotensive event during a dialysis session with subsequent recovery. There were no other ischemic or systemic embolic events. This is particularly remarkable given the single and short antiplatelet regimen with aspirin 100 mg/day for 3 months.

Only one patient with brain metastases presented a hemorrhagic stroke while being under treatment with aspirin. Six patients (11%) had major bleeding (BARC 3a) while being under treatment with aspirin. Notably, all these patients had a previous history of repeat bleeding.

Park et al. [10] conducted a study with 60 patients from which 77% (*n* = 47) completed 1-year follow-up, reporting 3% of mortality (unrelated to the device/procedure), 1.6% of TIA and 5% of minor bleeding.

### Study Limitations

The study was observational, non-randomized, and without control group. The sample size was relatively small, with no comparison group and with self-reporting of events. The bias related to the learning curve of the operators, which could have resulted in a higher incidence of complications at the beginning. Finally, larger registries with longer follow-up periods are required, as well as the evaluation of future randomized studies that compare LAAC with the LAmbre™ device to the standard treatment, as well as other LAAC devices.

## 5. Conclusions

This is a real-world registry which supports the feasibility, safety, and mid-term efficacy of LAAC with the LAmbre™ device, including complex LAA morphologies, in a high-complexity population with promising results. In addition, the combination of this device with a single and short antiplatelet therapy seems to be safe, showing remarkable mid-term performance regarding device thrombosis at 1-month and prevention of ischemic stroke at 1-year follow-up. Larger multicenter registries with long-term follow-up are required to confirm these results.

## Figures and Tables

**Table 1:** Baseline characteristics (*n* = 55).

Age (years)	75 ± 9.4
Age ≥ 75 years	33 (60)
Female sex	25 (45.5)
Hypertension	52 (94.5)
Diabetes	27 (49.1)
AF type	
-Paroxysmal	22 (40)
-Persistent	5 (9.1)
-Permanent	28 (50.9)
CHA_2_DS_2_-VASc score	4.6 ± 1.6
HAS-BLED score	3.9 ± 1.0
Heart failure	15 (27.3)
Coronary heart disease	18 (32.7)
Myocardial infarction	10 (18.2)
Previous coronary revascularization	16 (29.1)
Previous LVEF	57 ± 8.4
Previous stroke/TIA	21 (38.2)
Previous major bleeding	37 (67.3)
-Gastrointestinal	24 (43.6)
-Hemorrhagic stroke	9 (14.5)
-Intracranial	4 (7.3)
-Abdominal/retroperitoneal	1 (1.8)
Renal failure (GFR < 60 mL/min/m^2^)	41 (74.5)
-Stage 3a	10 (18.2)
-Stage 3b	9 (16.4)
-Stage 4	11 (20)
-Stage 5	11 (20)
Renal transplant	4 (7.3)
Hemodialysis	7 (12.7)
Labile INR	7 (12.7)
Contraindication for OAC	51 (92.7)
MDRD glomerular filtration rate (mL/min/m^2^)	35; (17–60)
Creatinine (mg/dL)	1.78; (0.98–2.91)
Hemoglobin (g/dL)	11.6 ± 1.8

AF, atrial fibrillation; GFR, glomerular filtration rate; INR, international normalized ratio; LVEF, left ventricular ejection fraction; MDRD, Modification of Diet in Renal Disease; OAC, Oral anticoagulation; TIA, transient ischemic attack. Values are expressed as *n* (%), mean and standard deviation, or median and interquartile range (25–75%).

**Table 2:** Procedural characteristics (*n* = 55).

Transseptal puncture	55 (100)
Combined procedure	14 (25.5)
-Diagnostic coronary angiography	11 (20)
-Coronary angioplasty	1 (1.8)
-Mitral valvuloplasty	2 (3.6)
Intraprocedural TEE	52 (94.5)
Intraprocedural ICE	7 (12.7)
Device size	24.9 ± 3.5
Change of initial device size	3 (5.4)
Procedure time (min)	96.6 ± 27.8
Fluoroscopy time (min)	17.6 ± 6.8
Contrast volume used (mL)	125.2 ± 49.4
Length of hospital stay (days)	1 (1–1)

ICE, intracardiac echocardiography; TEE, transesophageal echocardiography. Values are expressed as *n* (%), mean and standard deviation, or median and interquartile range (25–75%).

**Table 3:** Periprocedural complications (*n* = 55).

Procedural success	54 (98.2)
Device success	54 (100) *
Death	1 (1.8)
Vascular	2 (3.6)
-Femoral hematoma	1 (1.8)
-Femoral pseudoaneurysm	1 (1.8)
TIA/stroke	0 (0)
Major bleeding	1 (1.8)
-BARC 3a	1 (1.8)
Device embolization	0 (0)
Cardiac tamponade	2 (3.6)
Emergency surgery	1 (1.8)

TIA, transient ischemic attack. Values are expressed as *n* (%). * 100% of patients in whom device implantation was attempted.

**Table 4:** TEE follow-up 1 month after LAAC (*n* = 54).

N (% of total patients)	50 (92.6)
Device thrombosis	0 (0)
Significant leak (≥5 mm)	0 (0)
Non-significant leak (<5 mm)	12 (21.8)

TEE, transesophageal echocardiography. Values are expressed as *n* (%).

**Table 5:** Adverse events during 12-month follow-up (*n* = 54).

Overall mortality	1 (1.8)
Cardiovascular mortality	0 (0)
TIA/stroke	2 (3.6)
-Ischemic	1
-Bleeding	1
Major bleeding	6 (11)
-BARC 3a	5
-BARC 3c	1

TIA, transient ischemic attack. Values are expressed as *n* (%).

## Data Availability

The data presented in this study are available on request from the corresponding author. The data are not publicly available due to confidential patient information.

## Appendix A

**Table A1:** Learning curve for LAAC with the LAmbre™ device (*n* = 55).

Periprocedural Complications	First 27 Patients	Last 28 Patients
Death, *n* (%)	1 (3.7) *	0 (0)
Vascular complication, *n* (%)	2 (7.4)	0 (0)
TIA/stroke, *n* (%)	0 (0)	0 (0)
Major bleeding, *n* (%)	1 (3.7) *	0 (0)
Device embolization/need for SxI, *n* (%)	0 (0)	0 (0)
Cardiac tamponade, *n* (%)	2 (7.4)	0 (0)
*- Pericardiocentes*	1 (3.7)	0 (0)
*- Emergency surgery*	1 (3.7) *	0 (0)

LAAC, left atrial appendage closure; SxI, surgery intervention; TIA, transient ischemic attack. * Corresponds to the same patient.

**Table A2:** Learning curve for LAAC with the LAmbre™ device (*n* = 55).

	Type of Bleeding	Rebleeding	Same Type of Rebleeding	Time after LAAC (Months)	APT after LAAC	Baseline HAS-BLED	Baseline Renal Failure (GFR < 60 mL/min/m^2^)
1	Gastrointestinal	Yes	Yes	6	Aspirin	4	3b
2	Gastrointestinal	Yes	Yes	3	Aspirin	5	3b
3	Gastrointestinal	Yes	Yes	1	Aspirin	4	No
4	Unknown	Yes	No	1	Aspirin	5	3a
5	Unknown	Yes	Yes	9	None	6	2
6	Brain	Yes	No	1	Aspirin	4	5−HD

APT, antiplatelet therapy; HD, hemodialysis; LAAC, left atrial appendage closure.
